# *Lavandula pedunculata* Polyphenol-Rich Extracts Obtained by Conventional, MAE and UAE Methods: Exploring the Bioactive Potential and Safety for Use a Medicine Plant as Food and Nutraceutical Ingredient

**DOI:** 10.3390/foods12244462

**Published:** 2023-12-13

**Authors:** Ana A. Vilas-Boas, Ricardo Goméz-García, Manuela Machado, Catarina Nunes, Sónia Ribeiro, João Nunes, Ana L. S. Oliveira, Manuela Pintado

**Affiliations:** 1 CBQF—Centro de Biotecnologia e Química Fina—Laboratório Associado, Escola Superior de Biotecnologia, Universidade Católica Portuguesa, Rua Diogo Botelho 1327, 4169-005 Porto, Portugal; avboas@ucp.pt (A.A.V.-B.); rgarcia@ucp.pt (R.G.-G.); mmachado@ucp.pt (M.M.); asoliveira@ucp.pt (A.L.S.O.); 2 Centro de Investigación e Innovación Científica y Tecnológica—CIICYT, Universidad Autónoma de Coahuila, Saltillo 25280, Coahuila, Mexico; 3 Association BLC3—Technology and Innovation Campus, Centre Bio R&D Unit, Senhora da Conceição, 3045-155 Oliveira do Hospital, Portugal; catarina.nunes@blc3.pt (C.N.); sonia.ribeiro@blc3.pt (S.R.); joao.nunes@blc3.pt (J.N.)

**Keywords:** *Lavandula pedunculata*, rosmarinic acid, microwave-assisted extraction, ultrasound-assisted extraction, phenolic compounds, nutraceutical, food additive

## Abstract

Nowadays, plant-based bioactive compounds (BCs) are a key focus of research, supporting sustainable food production and favored by consumers for their perceived safety and health advantages over synthetic options. *Lavandula pedunculata* (LP) is a Portuguese, native species relevant to the bioeconomy that can be useful as a source of natural BCs, mainly phenolic compounds. This study compared LP polyphenol-rich extracts from conventional maceration extraction (CE), microwave and ultrasound-assisted extraction (MAE and UAE). As a result, rosmarinic acid (58.68–48.27 mg/g DE) and salvianolic acid B (43.19–40.09 mg/g DE) were the most representative phenolic compounds in the LP extracts. The three methods exhibited high antioxidant activity, highlighting the ORAC (1306.0 to 1765.5 mg Trolox equivalents (TE)/g DE) results. In addition, the extracts obtained with MAE and CE showed outstanding growth inhibition for *B. cereus*, *S. aureus*, *E. coli*, *S. enterica* and *P. aeruginosa* (>50%, at 10 mg/mL). The MAE extract showed the lowest IC_50_ (0.98 mg DE/mL) for angiotensin-converting enzyme inhibition and the best results for α-glucosidase and tyrosinase inhibition (at 5 mg/mL, the inhibition was 87 and 73%, respectively). The LP polyphenol-rich extracts were also safe on caco-2 intestinal cells, and no mutagenicity was detected. The UAE had lower efficiency in obtaining LP polyphenol-rich extracts. MAE equaled CE’s efficiency, saving time and energy. LP shows potential as a sustainable raw material, allowing diverse extraction methods to safely develop health-promoting food and nutraceutical ingredients.

## 1. Introduction

The *Lavandula* genus is prominent in the Mediterranean region, belonging to the botanical family Lamiaceae and encompassing 39 distinct species [1]. The *Lavandula* species possess intriguing economic significance as ornamental plants and for various industrial applications, including pharmaceutical, food, aromatherapy, perfumery, and cosmetics, mainly due to their valuable essential oils (EOs) [1]. *Lavandula pedunculata* (Miller) Cav. (LP) (known in Portugal as “rosmaninho-maior”) is common in the Iberian Peninsula [2]. With a widespread distribution, LP is considered the most resistant of all species of the *Lavandula* genus, growing in altitudes up to 1700 m, resisting annual temperature variations, and can reach up to 70 cm tall [3]. However, in Portugal, LP is scarcely used in phytotherapy, nor is it distilled to obtain EOs, due to a low ester content and a high amount of borneol and ketones [2]. It is used only in Portuguese folk medicine as infusions for anxiety and insomnia, the digestive system and as a therapeutic agent with antiseptic action for cleaning wounds [2,3].

Previous works have identified phenolic compounds such as rosmarinic acid, chlorogenic acids, salvianolic acid B, lithospermic acid A, apigenin and luteolin in extracts from the LP subspecies, *lusitanica* [3,4]. However, in comparison to EOs, the non-volatile fraction remains poorly described. Phenolic compounds have been shown to positively affect human health, lowering the risk of diabetes, cancer, cardiovascular disease, and neurological problems [5]. Recent studies have shown that LP extracts have potential through their antioxidant, anti-inflammatory, antiproliferative, antimicrobial, anti-tyrosinase and anti-cholinesterase activity [6]. In addition, LP extracts have also shown an antihyperglycemic effect using in vitro, ex vivo and in vivo methods. In vitro, LP aqueous extract inhibited pancreatic α-amylase and intestinal α-glucosidase enzymes [7]. Therefore, there is scientific evidence that LP can be helpful as a new potential source of natural BCs, mainly phenolic compounds, which could contribute to valorizing the existing biodiversity and resources of the Portuguese native species of LP. This represents an opportunity in the agricultural sector to promote the sustainable use of this aromatic plant and produce new sources of income for farmers, since the distillery industry does not use this plant species.

Based on a search using Web Of Science (selecting all databases; all of the time span until September 2023, and term: “*Lavandula pedunculata*” in Topic), a total of 44 articles in English were detected. In addition, a careful analysis was conducted to evaluate the articles’ titles and abstracts, and it was concluded that only nine research papers addressed the chemical and/or biological characterization of EOs or extracts from LP. Regarding studies about LP polyphenol-rich extracts, the most common extraction was conventional maceration extraction (CE) with non-green solvents (n-hexane, dichloromethane, and methanol) from non-renewable resources. Despite being widely used to extract phenolic compounds due to their strong extraction and dissolving capacities, these solvents and techniques have a negative environmental impact since they require high amounts of solvents and are energy- and time-consuming [8]. Furthermore, their use is associated with both environmental and human health risks. In 2015, the United Nations established the 2030 Agenda for Sustainable Development to overcome these drawbacks, which promotes the wise use of resources and energy. However, there is a pressing need to decrease the usage of organic solvents and improve the energy efficiency of procedures used to extract bioactive compounds (BCs) from plants or foods [9]. Therefore, green chemistry-based extraction methods emerge as a possible alternative to the CE method with organic solvents, given their advantageous attributes, namely decreasing or eliminating hazardous solvents and limiting the cost of solvent waste disposal [10]. Moreover, it allows safety extracts to be obtained that could be used in the food, pharmaceutical and cosmetic industries. Green techniques such as ultrasound-assisted extraction (UAE) and microwave-assisted extraction (MAE), among others, are being used for the recovery of various BCs from plants and food by-products. A recent study from Mansinhos et al. [4] used a combination of (UAE) and deep eutectic solvents to extract phenolic compounds from LP subsp. *lusitanica* Franco. However, a comparative study has not yet been reported between CE and green extractions such as UAE and MAE. Furthermore, to our knowledge, the use of the MAE technique for the phenolic compounds extraction from LP has not yet been evaluated.

The demand for plant-derived BCs and functional ingredients in the food industry has increased due to the current demand for “natural” products, free of harmful chemicals, which are expected to be safe and healthy [11]. The enzymatic browning of fruits and vegetables is one of the food industry’s most significant problems [12]. To inactivate the enzymes that cause browning (such as tyrosinase and other polyphenol oxidase enzymes (PPO)), the dipping technique employed frequently uses antioxidant agents and/or browning inhibitors, mainly in the IV gamma products [12,13]. Due to their wealth of phenolic compounds with strong antioxidant activity, LP-based extracts could be a natural option for the food industry to prevent browning. In addition, these natural extracts could also improve the shelf-life, reduce microbial contamination, and replace the use of synthetic food additives.

On the other hand, using natural extracts as food supplements to prevent and/or improve non-communicable diseases or enhance food’s nutritional value is on the rise. Natural BCs like flavonoids and phenolic acids have shown the ability to inhibit enzyme activity mainly involved in the mechanism of hypertension and diabetes (α-glucosidase and angiotensin-converting enzyme) [14,15]. Some studies have demonstrated that these natural extracts are similar to certain synthetic drugs in terms of activity; consequently, due to their safety and lower risk of side effects, the consumer’s interest is increasing. In the last decade, the market has seen an increase in the availability of natural food additives, functional foods, nutraceuticals and supplements. This industry is expected to grow to around USD 210 billion by 2026 [16]. Europe is gathering clinical evidence to determine the health benefits and toxicity of emerging natural BCs, as the food industry seeks sustainable products with various applications.

Therefore, the main objective of this research was to study the impact of the extraction methodology (MAE, UAE, and CE) on obtaining bioactive polyphenol-rich extracts from LP to select the best technique for producing a novel functional ingredient. The bioactive properties were explored regarding the antioxidant, antimicrobial, antidiabetic, and antihypertensive potential and for tyrosinase inhibition. In addition, cytotoxicity and genotoxicity evaluations were performed to validate the LP extracts’ safety.

## 2. Materials and Methods

### 2.1. Chemicals

The 1,5-Di-*O*-caffeoylquinic acid, 2-azinobis-3-ethylbenzothiazoline-6-sulphonic acid (ABTS), 2,2′-azo-bis-(2-methylpropionamidine)-dihydrochloride (AAPH), 2,2-diphenyl-1-picrylhydrazyl (DPPH), 2,5-dihydroxybenzoic acid, 3-*O*-caffeoylquinic acid, 4-*O*-caffeoylquinic acid, 4,5-Di-*O*-caffeoylquinic acid, 5-*O*-caffeoylquinic acid, acarbose, angiotensin-I converting enzyme (peptidyl-dipeptidase A, EC 3.4.15.1, 5.1 U/mg), ascorbic acid, caffeic acid, ferulic acid, fluorescein, food grade ethanol 99%, formic acid, gallic acid, rosmarinic acid, salvianolic acid B, sodium carbonate, trifluoroacetic acid, Trolox, α-glucosidase from *S. cerevisiae*, *p*-nitrophenyl-α-D-glucopyranoside, and dimethyl sulfoxide were purchased from Sigma-Aldrich (Sintra, Portugal). Luteolin-7-*O*-glucoside, apigenin-7-*O*-glucoside, and quercetin-3-*O*-glucoside were purchased from Extrasynthese (Genay, France). The tripeptide Abz-Gly-Phe(NO2)-Pro was purchased from Bachem Feinchemikalien (Bubendorf, Switzerland). Tris (tris (hydroxymethyl) aminomethane) was purchased from Fluka (Fluka Gmbh, Germany). Acetonitrile and methanol were purchased from Fischer Scientific (Oeiras, Lisbon, Portugal). Folin–Ciocalteu reagent and potassium persulfate were purchased from Merck (Algés, Lisbon, Portugal). Trifluoroacetic acid was purchased from VWR (Carnaxide, Lisbon, Portugal). Mueller–Hinton broth was purchased from Biokar Diagnostics (Patin, Paris, France).

### 2.2. Plant Material and Preparation of Extracts

#### 2.2.1. Plant Material

The studied *Lavandula pedunculata* (Mill.) Cav. (LP) consisted of young and old explants of stems, leaves and fruit harvested in 2019 on various locations, such as Gouveia, Fornos de Algodres, Vila Chã and Caldas da Cavaca, in the district of Viseu and Coimbra, Portugal, in March 2019. The raw material (LP) studied in this research work was dried in an oven at 40 °C for 48 h, immediately packed in a vacuum to preserve its properties and kept in a cool and dark place. The sample of LP to be studied encompassed its constituent parts: stems, leaves and fruit. They were reduced to particles of smaller dimensions (6 mm) in a conventional cutting mill (FRITSCH P-15; Idar-Oberstein, Birkenfeld, Germany) before the application of the extraction methodology.

#### 2.2.2. Extraction Methodologies

**Conventional Extraction with temperature (CE):** The experimental procedure was adapted from Dobros et al. [17]. Briefly, 3 g of dried LP was mixed with 30 mL of extraction solvent (ethanol 50% (*v*/*v*)) that was added into a round-bottom flask coupled with a condenser. The assembly was placed in a water bath at 65 °C for 1 h.

**Microwave-assisted Extraction (MAE):** The methodology was adapted from Leocádio [18]. Briefly, 3 g of LP was weighed into a glass beaker; then, 30 mL of ethanol 50% (*v*/*v*) was added. The solution was transferred to the microwave Teflon reactor to perform extraction by applying the Milestone START E microwave program with SK-12 rotor: 500 W of power, temperature increments of 16 °C/min up to 65 °C for 5 min, after reaching 65 °C, maintain for 15 min.

**Ultrasound-assisted Extraction (UAE):** The experimental procedure was adapted from Leocádio [18]. Briefly, 3 g of an LP plant was placed into a glass flask and 30 mL of ethanol (50% *v*/*v*) was added. The solution was sonicated in an ultrasonic bath (Fritsch; model LABORETTE 17) with a power of 120 W and a frequency range between 50 and 60 Hz for 15 min (without exceeding 25 °C).

After each extraction method, the extract was filtered through vacuum filtration, and then the filtrate was evaporated to remove ethanol using a rotary evaporator (40 °C; 175 mbar). The remaining aqueous extract was frozen at −80 °C and dried via freeze-drying (LyoQuest-85; Telstar, Portugal). Finally, the dry extracts were stored in polypropylene flasks and kept in a desiccator during the analysis. The extraction methods were performed in three independent extractions.

The extraction yield was calculated based on the amount of dry plant used to make the dried extracts (Equation (1)):

(1) $$Extractive\;Yield\left(\%\right)=\frac{Dried\;extract\;(g)}{weight\;of\;dried\;plant\;(g)}\times 100$$

### 2.3. Total Phenolic Content

The LP extracts’ total phenolic content (TPC) was determined using the Folin–Ciocalteu method [19]. Briefly, 80 µL of Folin–Ciocalteu reagent 10% (*v*/*v*) was added to 20 µL of extract (previously dissolved in distilled water), followed by 100 µL of sodium carbonate (7.5% (m/v)). The reagents were allowed to react in the dark at room temperature (25 °C) for 1 h. Later, absorbance was measured at 750 nm (Multiskan GO Microplate Spectrophotometer; Thermo Fisher Scientific Inc., Waltham, MA, USA) in a 96-well microplate (Nunc™; Thermo Fisher Scientific Inc., USA). Gallic acid was used as a standard for the calibration curve (0.010–0.125 mg/mL, y = 6.0796x + 0.1314, R^2^ = 0.999), and the results were expressed as milligrams equivalent of gallic acid per gram of dry extract (mg GAE/g DE). Three independent analyses were performed in each triplicate extract obtained for each methodology.

### 2.4. Phenolic Compounds Identification by LC-ESI-QqTOF-HRMS

The polyphenol-rich LP extracts were dissolved in ultrapure water at 10 mg/mL for further analysis by LC-ESI-UHR-QqTOF-MS, according to the method reported by Vilas-Boas et al. [19] Briefly, the separation was performed in a UHPLC UltiMate 3000 Dionex (Thermo Scientific, Waltham, MA, USA), coupled to an ultrahigh-resolution, Qq-time-of-flight (UHR-QqTOF) mass spectrometer with 50,000 full-sensitivity resolution (FSR) (Impact II; Bruker Daltonics, Bremen, Germany). The separation was accomplished with an Acclaim RSLC 120 C18 column (100 mm × 2.1 mm, 2.2 μm) (Dionex, Sunnyvale, CA, USA). The injection volume was 5 μL. The mobile phases consisted of (A) 0.1% aqueous formic acid and (B) acetonitrile with 0.1% of formic acid and the gradient elution conditions were: 0 min, 0% B; 10 min, 21.0% B; 14 min, 27% B; 18.30 min, 58%; 20.0 min, 100%; 24.0 min, 100%; 24.10 min, 0%; 26.0 min, 0% at a flow rate of 0.25 mL/min. Parameters for MS analysis were set using negative ionization mode with spectra acquired over a range from m/z 20 to 1000. The parameters were as follows: capillary voltage, 3.0 kV; drying gas temperature, 200 °C; drying gas flow, 8.0 L/min; nebulizing gas pressure, 2 bar; collision RF, 300 Vpp; transfer time, 120 μs; and prepulse storage, 4 μs. Post-acquisition internal mass calibration used HCOONa clusters, delivered by a syringe pump at the start of each chromatographic analysis. High-resolution mass spectrometry was used to identify the compounds. The elemental composition for the compound was confirmed according to accurate mass and isotope rate calculations designated mSigma (Bruker Daltonics, Billerica, MA, USA). The accurate mass measurement was within 5 mDa of the assigned elemental composition, and mSigma values of <20 provided confirmation. Compounds were identified based on their accurate mass [M-H]^−^. One independent analysis was performed in each triplicate extract obtained for each methodology.

### 2.5. Phenolic Compounds Quantification by HPLC–DAD

The quantitative profiling of phenolic compounds in polyphenol-rich LP extracts (previously dissolved in ultrapure water at 20 mg/mL) was performed using a Waters Alliance e2695 separation module system interfaced with a photodiode array UV/Vis detector 2998 (PDA 190–600 nm; Waters, Mildford, MA, USA). The separation occurred in a reversed-phase C18 column (ZORBAX Eclipse XDB-C18, 80A°; 4.6 × 250 mm; 5 μm; Agilent, Santa Clara, CA, USA) at 25 °C. The mobile phase and the gradient elution used were prepared according to Vilas-Boas et al. [19]. The injection volume and the flow rate were 20 µL and 1 mL/min, respectively. Data acquisition and analysis were carried out using Software Empower 3. The detection was performed at 280, 320, 350, and 360 nm, and the phenolic compound identification was performed by comparing the retention time and absorbance spectra with pure standards. The quantification was performed using the calibration curves’ interpolation, and the results were expressed as milligrams per gram of dry extract (mg/g DE). Three independent analyses were performed in each triplicate extract (CE, MAE and UAE) obtained for each methodology.

### 2.6. Antioxidant Activity

#### 2.6.1. ABTS Method

The CE, UAE and MAE extracts described in Section 2.2.2 were evaluated for their antioxidant capacity against the ABTS free radical, as described by Vilas-Boas et al. [19]. The ABTS stock solution was prepared by mixing ABTS aqueous solution (7 mmol/L) with potassium persulfate aqueous solution (2.45 mmol/L) and kept for 16 h in the dark at 25 °C. On the analysis day, the ABTS stock solution was diluted with water to an absorbance of 0.700 ± 0.020 at 734 nm (ABTS working solution). After that, 15 μL of sample (previously dissolved in distilled water) was allowed to react in the darkness at room temperature (25 °C) with 200 μL of ABTS working solution and the absorbance was read at 734 nm 6 min precisely after initial mixing in a 96-well microplate (Nunc™; Thermo Fisher Scientific Inc., USA). A blank was taken with distilled water (A0). The inhibition percentage of the sample was calculated using Equation (2) and compared with the ascorbic acid standard calibration curve (0.0088–0.0880 mg/mL; r^2^ = 0.999). The assay was realized with the multidetector plate reader Synergy H1 (BioTek Instruments, Winooski, VT, USA) controlled by the Gen5 BioTek software version 3.04.

The results were expressed as milligrams of ascorbic acid equivalent per gram of dry extract (mg AAE/g DE). Three independent analyses were performed in each triplicate extract obtained for each methodology.

#### 2.6.2. DPPH Method

The DPPH assay was carried out according to the procedure described by Vilas-Boas et al. [19]. Briefly, 175 µL of DPPH work solution (60 µM) was allowed to react with 25 µL of extract (previously diluted in distilled water) in a 96-well microplate (Nunc™; Thermo Fisher Scientific Inc., USA) for 30 min in darkness. Then, the absorbance was measured at 515 nm (multidetection plate reader Synergy H1; BioTek Instruments, Winooski, VT, USA), and a blank was taken with distilled water (A0). The inhibition percentage of the sample was calculated using Equation (2), and Trolox was used as a standard to prepare a calibration curve (0.0075–0.0750 mg/mL; r^2^ = 0.999). The results were expressed as milligrams of Trolox equivalent per gram of dry extract (mg TE/g DE). Three independent analyses were performed in each triplicate extract obtained for each methodology.

(2) $$\%\;Inhibition=\frac{{Abs}_{A0}-{Abs}_{sample}}{{Abs}_{A0}}\times 100$$

Abs_A0_ is the absorbance of blank, and Abs_sample_ is the absorbance of the reaction between the sample and the radical after the incubation.

#### 2.6.3. ORAC Method

An oxygen radical absorbance capacity (ORAC) assay was carried out following the methodology described by Coscueta et al. [20]. The reaction was carried out in a 75 mM phosphate buffer (pH 7.4), with a final reaction volume of 200 µL. The sample (20 µL), previously dissolved in distilled water, and fluorescein solution (FL) (120 µL; 70 nM, final concentration in well), was added to the well of the black 96-well microplate (Nunc, Denmark). Each plate experiment also included a calibration curve (1–8 µM of Trolox, final concentration in well) and a blank (FL + AAPH + phosphate buffer). The mixture was preincubated at 37 °C for ten minutes. After that, the AAPH solution (60 µL; 12 mM, final concentration in well) was added rapidly using a multichannel pipet. The fluorescence was recorded at intervals of 1 min for 80 min. A multidetector plate reader (Synergy H1; Biotek, Winooski, VT, USA) with 485 nm excitation and 520 nm emission filters was used. The equipment was controlled by the Gen5 Biotek software version 3.04. The results were expressed as milligrams of Trolox equivalent/g of dry extract (mg TE/g DE). Three independent analyses were performed in each triplicate extract obtained for each methodology.

### 2.7. Antimicrobial Activity

#### 2.7.1. Bacterial Strains

The Gram-negative bacteria used were *Escherichia coli* (ATCC (American type culture collection) 25922), *Salmonella enterica serovar Enteritidis* (ATCC 13076) and *Pseudomonas aeruginosa* (ATCC 278539) while *Listeria monocytogenes* (NCTC (National Collection of Type Cultures) 10357), *Bacillus cereus* (NCTC 2599) and *Staphylococcus aureus* (ATCC 25923) were the Gram-positive bacteria used. These bacteria were cultured on Mueller–Hinton broth (MHB) medium and incubated at 37 °C overnight. The inoculum test concentration after this period should be 10^8^ CFU/mL.

#### 2.7.2. Growth Inhibition Curves

The dry extracts of LP were re-dissolved in MHB at three different concentrations (10, 5 and 2.5% (*w*/*v*)) and sterilized by filtration through a 0.22 µm filter (FriLabo—Maia, Portugal). The growth inhibition curves were determined using the broth microdilution assay, following the standards for antimicrobial susceptibility testing provided by the Clinical and Laboratory Standards Institute (CLSL) [21]. At the time of testing, each fresh overnight culture of bacteria (prepared in Section 2.7.1) was adjusted spectrophotometrically (optical density (OD) at 625 nm) between 0.08 and 0.13, representing a concentration of an inoculum of 1 × 10^8^ CFU/mL. Afterwards, each well of a sterile 96-well microplate was filled with a total volume of 200 µL containing approximately 10^6^ CFU/mL of test bacteria (1% (*v*/*v*) inoculum) and variable concentrations of each extract prepared. A positive control containing only MHB inoculated at 1% (*v*/*v*) and two negative controls: (i) containing only sterilized medium and (ii) containing only sterilized extract, were also tested. The OD at 625 nm was assessed for a period of 24 h at 37 °C (1 h intervals) by using a microplate reader (Multiskan GO Microplate Spectrophotometer; Thermo Scientific, Thermo Fisher Scientific Inc., Waltham, MA, USA). The increase in OD was considered a direct consequence of bacterial growth. Each condition was assayed in triplicate. An inhibition percentage [22] was calculated using the following Equation (3):

(3) $$Inhibition\;Percentage\left(\%\right)=\frac{OD\;control-OD\;bacteria}{OD\;control}\times 100$$

OD control bacteria and OD bacteria represent the OD (at 625 nm) after 24 h of incubation of the control bacteria in the growth medium and in the presence of the LP extracts, respectively.

### 2.8. Tyrosinase Inhibition Assay

The capability of LP extracts to inhibit tyrosinase enzyme was assessed with a colorimetric tyrosinase inhibitor screening kit ( ab204715, Abcam). Briefly, a mixture of extracts previously diluted in the buffer (20 µL) and tyrosinase solution (50 µL) was incubated in a 96-well microplate at 25 °C for 10 min. Then, 30 μL of substrate solution was added, and the absorbance was continuously read at 510 nm in a microplate reader (Synergy H1; BioTek Instruments, Winooski, VT, USA). Data were recorded every 2 min time interval for 30 min. An inhibition control (IC) was performed with Kojic acid (0.021 mg/mL). A positive control for the enzyme was performed with a mixture of enzyme and substrate in the presence of buffer. The average was made for reading duplicates. Two time points (T1 and T2) were chosen in the linear range of the plot and obtained the corresponding values for absorbance (A1 and A2). The slope was calculated for all samples (S), inhibition control (IC) and enzyme control (EC) by dividing the net ΔA (A2 − A1) values with the time ΔT (T2 − T1). The percentage inhibition was calculated using Equation (4):

(4) $$Tyrosinase\;inhibition\left(\%\right)=\frac{{Slope}_{EC}-{Slope}_{S}}{{Slope}_{EC}}$$

### 2.9. α-Glucosidase Inhibition Assay

The α-glucosidase inhibition was determined using a colorimetric-based method according to Know et al. [23]. Firstly, the dry extracts were dissolved and diluted in the phosphate buffer (pH 6.9; 0.1 M), and the final concentrations tested were between 5.0 and 1.25 mg/mL. A total of 50 µL of extract solution and 100 µL of phosphate buffer (pH 6.9; 0.1 M) containing α-glucosidase (1.0 U/mL) were incubated in 96-well plate at 25 °C during 10 min. After that, 50 µL of a 5 mM *p*-nitrophenyl-α-D-glucopyranoside solution prepared in phosphate buffer (pH 6.9; 0.1 M) was added to each well with a multichannel pipettor and the absorbance was read. The plate was incubated at 25 °C for 5 min, and absorbance readings were recorded at 405 nm with a microplate reader (Synergy H1, BioTek Instruments, Winooski, VT, USA). As a negative control, 50 μL of buffer solution replaced the extracts. Acarbose was used as a positive control at a 5 mg/mL concentration. A blank without the enzyme (100 μL of 0.1 mol/L phosphate buffer instead) was performed for each sample. The α-Glucosidase inhibitory activity was expressed as inhibition (%) and calculated using Equation (5):

(5) $$\alpha \text{-}glucosidase\;inhibition\left(\%\right)=\frac{{\Delta Abs}_{C}-\Delta {Abs}_{S}}{\Delta {Abs}_{C}}$$

where ΔAbs_c_ is the variation in absorbance of the control, and ΔAbs_s_ is the variation in the samples’ absorbance.

### 2.10. Angiotensin-Converting Enzyme-I Inhibition Assay (iACE)

The in vitro iACE assay was conducted according to the procedure outlined by Coscueta et al. [24]. A commercial angiotensin-I converting enzyme (EC 3.4.15.1, 5.1 U/mg) was diluted in 5 mL of a glycerol solution in 50% ultra-pure water. The stock solution was then diluted 1:24 with a 150 mM Tris buffer solution, pH 8.3, containing 1 μM of ZnCl_2_, reaching a 42 mU/mL concentration in the final reaction solution. Then, 40 μL of the working solution of ACE was added to each well of the reaction microplate and then adjusted to 80 μL by adding ultra-pure water for the control and the sample to be analyzed. The enzymatic reaction was initiated by adding 160 μL of a 0.45 mM solution of ABz-Gly-Phe(NO_2_)-Pro, dissolved in 150 mM Tris buffer (pH 8.3), containing 1.125 M NaCl, mixing immediately and incubating at 37 °C. The microplate was automatically shaken before the first reading, and the fluorescence generated was measured for 30 min. The measurements were completed with the Synergy H1, (BioTek Instruments, Winooski, VT, USA) plate reader with excitation filters at 350 nm and emission at 420 nm. Nunc^TM^ black 96-well polystyrene microplates (ThermoScientific, Waltham, MA, USA) were used for the assay. Each extract was evaluated in triplicate, and the iACE was expressed as the concentration capable of inhibiting 50% of the ACE activity (IC_50_). Non-linear modelling was applied to determine the IC_50_, and the results were expressed as mg/mL to inhibit 50% of the enzymatic activity.

### 2.11. Cytotoxicity

#### 2.11.1. Cell Line Growth Conditions

Human Caucasian colon carcinoma epithelial cells, Caco-2 (86010202 European Collection of Authenticated Cell Cultures) cells were grown in Dulbecco’s modified Eagle’s medium (Lonza, Basel, Switzerland) supplemented with 10 % (*v*/*v*) heat-inactivated fetal bovine serum (Biowest, Nuaillé, France), 1% (*v*/*v*) penicillin-streptomycin-fungizone (Lonza, Basel, Switzerland) and 1% (*v*/*v*) non-essential amino acids 100× (Lonza, Basel, Switzerland). All cells were incubated at 37 °C in a humidified atmosphere with 5% CO_2_.

#### 2.11.2. Cytotoxicity Assay

The cytotoxicity evaluation was performed according to the ISO 10993-5:2009 standard [25], Cells were grown to 80–90% confluence, detached using TrypLE Express (ThermoScientific, Waltham, MA, USA), and seeded at 1.0 × 10^5^ cells/mL into 96-well tissue cultured plates (Nunclon ThermoScientific, Waltham, MA, USA) and allowed to adhere for 24 h. Afterwards, the media were carefully removed and replaced with media supplemented with LP extracts at 4.8–0.4 mg/mL concentrations. DMSO at 40% in culture media was used as the death control, and plain culture media was used as the growth control. After the incubation time, Presto Blue (ThermoFisher, Waltham, MA, USA) was added to each well and incubated at 37 °C in the darkness for 1 h. After this period, fluorescence (Ex: 560 nm; Em: 590 nm) was measured using a microplate reader (Synergy H1; Biotek Instruments, Winooski, VT, USA). All assays were performed in quintuplicate.

### 2.12. Mutagenicity Evaluation—AMES Assay

AMES assay was performed in accordance with OECD guideline 471 [26], using the MOLTOX^®^ Salmonella Mutagenicity Assay Kit (reference number: 31-110.2; Trinova Biochem GmbH, Giessen, Germany), according to the manufacturer’s instructions. *Salmonella typhimurium* TA 98 was previously inoculated and incubated at 37 °C and held stationary overnight for this assay. After that, the inoculum was set in an orbital (130 rpm at 37 °C) until a density of 1–2 × 10^9^ bacteria/mL (approximately 1.0–1.4 at OD 650 nm) was reached. Samples were tested at 1 mg/plate, 0.5 mg/plate and 0.125 mg/plate, corresponding to 10, 5 and 1.25 mg/mL, respectively. Two controls were assessed: one with chemical control capable of inducing bacterial mutation Daunomycin and 2-Aminoanthracene) and another with water instead of the extract (solvent control). The assay was placed in a 37 °C incubator for approximately 48 h. The assays were performed with and without metabolic activation (S9). Two plates per three separate experiments were assayed for each concentration tested and for positive and negative controls. According to the kit instructions, a positive result (genotoxic effect) should be at least 2.5 times in a treatment group concerning the corresponding negative control (solvent control).

### 2.13. Statistical Analysis

The results in Section 3 are presented as the average ± standard deviation of three independent extractions (n = 3). Shapiro–Wilk test was used to evaluate the normality of data distribution, Levene’s test was used for homogeneity of variances and the one-way analysis of variance (ANOVA) was used to examine the significance of the differences between the extracts. For ANOVA, the null hypothesis (H_0_) (means are equal) was rejected when the differences between means showed a *p* < 0.05. In this case, multiple comparisons were examined using Tukey’s post-hoc test (homogeneity of variance was assumed) at the *p* < 0.05 significance level. The statistical analysis was carried out entirely with SPSS version 23.

## 3. Results

Three LP polyphenol-rich extracts were prepared from dried LP using three different techniques: CE, UAE and MAE. The aim was to assess the effects of each technique and identify the optimal method for deriving a nutraceutical or health-enhancing food ingredient from a traditionally underexplored Portuguese medicinal plant. While these *Lavandula* species are seldom used for the commercial distillation of EOs, most studies describe their health benefits and applications. However, the potential of its polar extracts rich in phenolic compounds remains relatively unexplored. Therefore, the hydroethanolic polyphenol-rich extracts were further examined for their phenolic compounds and bioactivities using chemical in vitro assays. Additionally, the cytotoxicity and mutagenicity of the polyphenol-rich LP extracts were evaluated to ensure their safe application.

### 3.1. Extractive Yield

The primary extraction objective was to obtain the desired BCs with high extraction yields while reducing the concentration of unwanted compounds, such as proteins and sugars, which could affect the stability and quality of the final extract. The extraction efficiency may be affected by several factors, the most important of which are the solvent, mass-to-solvent ratio, temperature, and pH [27]. The variables regarding the type of solvent and ratio were fixed because one of the main goals of this research study was to develop extracts by applying distinct techniques and evaluating their impact. For this, an ethanol solution at 50% (*v*/*v*) was chosen because it is one of the most frequently used solvents; ethanol is food grade and is safe for the consumer even if not completely removed from the final ingredient; it is environmentally friendly and it can be reused in the process.

The total extraction yield (*w*/*w*, %) of the LP polyphenol-rich extracts obtained by different extraction techniques is shown in Table 1. The use of the CE technique resulted in a higher extraction yield (23.91 ± 2.00%, *w*/*w*) (*p* < 0.05), while the remaining green extraction techniques (UAE and MAE) recovery yield was about 16–17% *w*/*w* (*p* > 0.05). Nonetheless, it is important to emphasize that achieving a higher extractive yield does not necessarily equate to the extraction of a greater quantity of phenolic compounds, because specific extraction methods or conditions might favor the extraction of other components such as sugars, organic acids, minerals, and proteins. It is possible that the highest yields were caused by the high temperature, as the plant:solvent ratio, solvent type, and moisture content were the same for all extraction procedures. High temperatures (65 °C) were applied in the CE and MAE extractions. It influenced the extraction efficiency since a higher temperature promotes increased solubility and diffusion into the solvent, leading to a higher mass transfer rate [28]. According to research from Lezoul et al. [29], using high temperatures associated with CE helps access a better yield and, consequently, higher TPC. However, this effect cannot be generalized since it strongly depends on the type of compound extracted, the binomial time:temperature used and the heating type. While, statistically, the yield is lower when employing MAE, it is important to note that this technique is a high-temperature extraction method with significantly reduced extraction times, resulting in lower energy consumption than CE. UAE is reported to be a technique that achieves high yields quickly since it is especially good at breaking the cell walls, leading to the release of BCs. However, the lower yield obtained with UAE in this study could be attributed to the fact that high temperatures were not used in this methodology. Nevertheless, the results obtained are in line with those reported by Costa et al. [30], which brought extraction yields of 19.4% for LP extracts using CE (without use of high temperature and ethanol 50% (*v*/*v*)).

### 3.2. Total Phenolic Content

Phenolic compounds are widely distributed in plants such as the *Lavandula* species and are known for their antioxidant properties; however, in recent years, they have been linked to other properties such as enzyme inhibition involved in diabetes and hypertension, and fruit and vegetable browning [31]. Table 2 shows the results of the total phenolic content (TPC), which varied between 183.1 ± 4.9 and 183.7 ± 17.8 mg GAE/g DE. Despite the absence of statistically significant differences (*p* > 0.05), it is worth noting that methods involving high temperatures, such as CE and MAE, exhibited a higher total phenolic content (TPC), whereas UAE yielded the lowest value. These values follow the extraction yields obtained. As previously mentioned, the temperature seems to have an important impact on the extraction of phenolic compounds. The warmth may cause the plant tissues to soften and the bindings between polysaccharides and/or proteins and phenolic compounds to weaken, which could cause disruption and migration to the solvent [32]. However, because homogenous heating might be achieved more quickly and with less energy usage thanks to the energy transmission mechanism, MAE heating has been found to offer greater benefits than convection. In addition, UAE emerges as an excellent alternative to establishing an environmentally friendly extraction method. It involves physical and chemical phenomena completely different from those applied with MAE and CE with convectional heating. The ultrasound waves induce the cavitation forces, which induce the explosive collapse of bubbles and generate pressure causing plant tissue rupture and improving the release of intracellular compounds into the solvent. However, in this study, the UAE was less efficient than CE in extracting phenolic compounds. On the contrary, a recent study reported that UAE (15 min with 80% ethanol) was more efficient than CE [4]. However, the UAE took place at 50 °C. In addition, Dobros et al. [17] reported a higher yield in TPC (30.25 mg GAE/g dry weight (DW) with UAE (ethanol 50% for 30 min) than CE (ethanol 50% for 30 min) (28.01 mg GAE/g DW). It is important to highlight that both studies reported a TPC of approximately 30 mg GAE/g DE, while our LP extract presents a TPC of 181.4 mg/g DE. To the best of our knowledge, no one has ever published a comparison of these two green extraction methods with CE for extracting phenolic compounds from LP. There are only studies with CE (using polar and non-polar solvents), UAE extraction (with methanol, ethanol 80% (*v*/*v*) and water), and UAE in combination with deep eutectic solvents (DES). Based on the results from Baptista et al. [33], the higher value was obtained with a CE with methanol for 24 h (1040.3 ± 17.8 mg GAE/g), while the CE with 100% water allowed a TPC of about 675 mg GAE/g. Considering the last value, our results, regardless of the extraction method, were about 3.4-fold times less than reported in Baptista et al.’s study. However, our extraction time was lower than in that study. On the other hand, LP extracts obtained with CE using ethanol 80% (*v*/*v*) showed a TPC value ranging between 56.1 and 136 mg/g DE [3], while LP extracts obtained with CE (1 h; 200 rpm; 50 °C) showed a TPC value lower than 30 mg/g DE [4].

Overall, TPC represented about 18% of the total LP polyphenol-rich extract. Therefore, other compounds were extracted, including sugars, minerals, organic acids, fibers and proteins.

### 3.3. Phenolic Compounds Profile and Quantification

**Figure 1:**
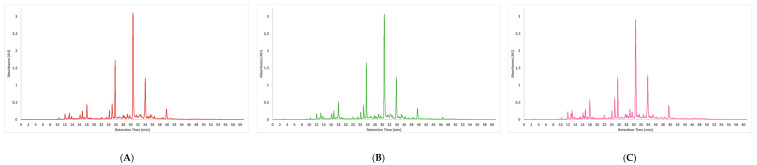
Chromatograms of phenolic compounds (HPLC-DAD) at 320 nm for CE (**A**), UAE (**B**) and MAE (**C**) extraction. Peaks: 1: 2,5-Dihydroxybenzoic acid; 2: 3-*O*-caffeoylquinic acid; 3: 5-*O*-caffeoylquinic acid; 4: 4-*O*-caffeoylquinic acid; 5: caffeic acid; 6: 1,5-Di-*O*-caffeoylquinic acid; 7: ferulic Acid; 8: luteolin-7-*O*-glucoside; 9: luteolin derivative; 10: 4,5-Di-*O*-caffeoylquinic acid; 11: apigenin-7-*O*-glucoside; 12: apigenin derivative; 13: quercetin-3-*O*-glucoside; 14: rosmarinic Acid; 15: salvianolic acid B: lithospermic acid.

A total of thirty phenolic compounds and seven organic acids were identified using the high-resolution mass spectrometry technique (Table 3). The identification of these compounds led to their distribution into three structurally related classes, i.e., hydroxycinnamic acids (14 compounds), hydroxybenzoic acids (6 compounds) and flavonoids (9 compounds). The identification of all proposed compounds presented a good mass accuracy (<2 mDa), increasing the confidence of the predicted identification. In detail, only five studies were found in the literature that identified phenolic compounds from LP extracts using mass spectrometry without differences (Figure 1). Two of these studies used plant material grown in Portugal [3,4]. The remaining three articles used LP grown in Morocco [7,34] and Turkey [35]. The most abundant compounds found in all the LP extracts were the hydroxycinnamic acids (50%), which agrees with the literature available for LP [3,4] and other *Lavandula* species [6]. Nevertheless, previous studies from Lopes et al. [3] identified only thirteen phenolic compounds, nine hydroxycinnamic acids and four flavonoids. Conversely, a study conducted by Mansinhos et al. [4] discovered twenty-three phenolic compounds in extracts obtained using UAE in conjunction with either 80% (*v*/*v*) ethanol or deep eutectic solvents. Similar to our findings, over 50% of the compounds were identified as hydroxycinnamic acids. Chlorogenic acids (CGAs) were widely distributed in *Lavandula* species. The main compounds were 3-*O*-caffeoylquinic acid, 5-*O*-caffeoylquinic acid and 4-*O*-caffeoylquinic acid, with a molecular ion [M-H]^−^ at 353, which was consistent with the molecular formula C_16_H_18_O_9_ and showed the typical fragment of loss as quinic acid (m/z 191). Moreover, another important class of CGAs was found: diCQAs (1,5-Di-*O*-caffeoylquinic acid and 4,5-Di-*O*-caffeoylquinic acid) with a [M-H]^−^ at 515 and a fragment m/z at 353 representing the caffeoylquinic acid.

In addition, the presence of caffeic acid is characteristic of the family *Lamiaceae* and is responsible for its potential bioactivity. Ferulic acid and isoferulic acid were identified in all extracts, which agrees with the report by Mansinhos et al. [4] concerning extracts obtained with UAE. However, this is the only study with LP that reports this compound. This can be explained by the fact that this compound is strongly linked to the cell wall and requires high temperatures or, for example, ultrasonic waves to extract it. Caffeic acid [M-H]^−^ at 179 was detected in the extracts, which was also reported in all the studies about LP. Within hydroxycinnamic acids, rosmarinic acid (RA) claims to be the most representative of the Lavandula genus. RA is an ester of caffeic acid with an [M-H]^−^ at 359. Therefore, for its correct identification, a m/z fragment should be at 179 (caffeic acid loss) and 161 (salvianic acid A loss). Other important compounds in the *Lavandula* genus belonging to this class are lithospermic acid A and salvianolic acid (also known as lithospermic acid B). The difference between RA and lithospermic acid A is the RA first lost the caffeic acid or the salvianic acid from [M-H]^−^, while the lithospermic acid A first lost CO_2_ from [M-H]^−^ m/z 537 (which corresponds with the m/z fragment 493) and then lost caffeic acid (fragment m/z 313) and salvianic acid A (fragment m/z 295). In the case of salvianolic acid B, it successively lost two molecules of caffeic acid or salvianic acid A loss, resulting in the fragment ion at m/z 537, m/z 519, m/z 339 and m/z 321 [36].

Hydroxybenzoic acids are secondary metabolites responsible for signaling in the defense of plants to pathogens, and high levels are related to systemic infections. Furthermore, they have interesting biological properties making them attractive as nutraceuticals or functional food additives [37]. Despite 20% of the phenolic compounds detected in LP polyphenol-rich extracts being classified as hydroxybenzoic acids, none of these individual compounds have been identified explicitly in LP of Portuguese origin. However, extracts obtained with UAE showed gallic and vanillic acid in their composition [4]. On the other hand, Boutahiri et al. [7,34] also identified protocatechuic acid and gentisic acid in methanolic and aqueous extracts from LP grown in Morocco. Therefore, this study represents a groundbreaking discovery as it is the first report of a profusion of compounds belonging to this class being identified in LP, grown in a Portuguese geographical origin.

Flavonoid subclasses observed in the genus *Lavandula* mainly include flavones, flavonols, and anthocyanins. However, in LP extracts obtained using CE, MAE, and UAE, flavones (luteolin and apigenin) and one flavonol (quercetin 3-*O*-glucoside) were predominantly observed. According to the previous literature studies, compounds such as luteolin-7-*O*-glucoside, luteolin-7-*O*-glucuronide, luteolin-*O*-hexosyl-glucuronide are the most representative in LP. In agreement with these studies, hydroethanolic and aqueous extracts obtained with LP showed these three flavones. However, in addition to reporting these compounds, Mansinhos et al. [4] also reported the presence of apigenin-7-*O*-glucoside and apigenin in extracts obtained with UAE, which aligns with the results observed in our extracts. It is worth noting that although apigenin-8-*O*-glucoside and quercetin 3-*O*-glucoside have already been reported in *L. stoechas* and other *Lavandula* species, this is the first time that the existence of these flavonoids have been reported in LP.

Sixteen phenolic compounds were quantified by HPLC-DAD in comparison with commercial standards (Table 4). The polyphenol-rich LP extracts (MAE, UAE, and CE) presented a similar profile (Figure 1), as previously discovered using mass spectrometry analysis. RA was the most abundant compound found in all extracts, followed by salvianolic acid B. The values range from 58.68 ± 1.42 to 42.27 ± 1.92 mg/g DE and 43.19 ± 1.09 to 40.09 ± 1.61 mg/g DE, respectively. Applying two green extractions showed less RA recovery than the CE (*p* < 0.05). This follows the study of Sik et al. [38], which compares different extraction methods to recovery RA from six Lamiaceae plants. However, these authors reported that the RA yield was lower in MAE when the extraction time was increased from 5 mins to 30 mins using high temperatures (80 °C), possibly due to the lower thermal stability of hydroxycinnamic acid derivatives. Hence, the high temperature disrupts the C-C bonds [39]. This is because the amount of energy converted to heat in the dielectric material is controlled by microwave power, which is correlated with the extraction temperature. Nevertheless, MAE total extraction time was four times less, reducing the energy expenditure. Regardless of salvianolic acid B extraction, the MAE showed the same efficiency as the CE (*p* > 0.05), but the UAE showed lower efficiency. However, this result could be reversed if UAE was optimized using a response surface model for the simultaneous higher yield extraction of salvianolic acid B and RA, which are the main phenolic compounds present in this plant.

Overall, the results obtained with LP extracts are in accordance with a study by Lopes et al. [3], who reported that RA and salvianolic acid B were the major compounds among all thirteen compounds identified in LP. A paper by Costa et al. [30] also reported RA as the most abundant compound in the water:ethanol extract of *L. pedunculata* subsp. lusitanica, but the concentration was smaller in comparison with our results. Moreover, in that study, only six different phenolic compounds were detected (3-*O*-caffeoylquinic acid, 4-*O*-caffeoylquinic acid, 5-*O*-caffeoylquinic acid, RA, luteolin and apigenin). A more recent study by Mansinhos et al. [4] used UAE to improve the recovery of phenolic compounds from LP subsp. lusitanica, and identified twenty-four phenolic compounds, with RA, ferulic acid and salvianolic acid B as the major compounds. Besides RA and salvianolic acid B, chlorogenic acid, caffeic acid, and Luteolin-7-*O*-glucoside were also identified in our LP extract from UAE, but in higher amounts.

The highest content of RA was found in the extract obtained by CET, followed by UAE and MAE (*p* < 0.05). Recent studies of Caleja et al. [40], comparing the same three techniques to extract RA from *Melissa officinalis* L. (Lamiaceae family), showed that UAE proved to be the most effective method than MAE to recover RA. However, Ince et al. [41] demonstrated that MAE was more efficient in extracting RA than CE and UAE. These discrepancies in the results may be because in the studies mentioned, the proportion of the water:ethanol in the extraction solvent was optimized based on response surface models. The same efficiency of UAE was observed for 2,5-dihydroxybenzoic acid and 4,5-*O*-Dicaffeoylquinic acid (*p* < 0.05).

In contrast, CGAs, ferulic acid, salvianolic acid B, lithospermic acid A, and flavonoids were found in greater quantities in the extracts obtained by MAE (*p* < 0.05). UAE showed a lower concentration (*p* < 0.05) of flavonoids; this can be justified because this method, despite applying acoustic cavitation due to the propagation of mechanical waves, did not reach high temperatures, as was the case with CE and MAE. Therefore, the MAE extract showed higher amount of flavonoids (22%) in comparison to CE (15%) and UAE (12%). Conversely, this extract contained fewer phenolic acids than the extracts of UAE (88%) and CE (85%). Regarding caffeic acid and 1,5-Di-*O*-caffeoylquinic acid, the lowest content was found in the CET extract (*p* < 0.05) and no significant differences (*p* > 0.05) were found between UAE and MAE.

RA was the most abundant compound found in the extracts, followed by ferulic acid and salvianolic acid B. These results are following results previously obtained for other *Lavandula* species. Lopes et al. [5] identified thirteen compounds in *L. pedunculata* (Mill.) Cav., being RA (50.9 mg/g DE) and salvianolic acid B (44.3 mg/g DE) the major compounds (in LP grown in Portalegre). However, in same study LP from Bragança showed only 7.5 mg/g DE of RA and 8.7 mg/g DE of salvianolic acid B. Similarly, Costa et al. [30] obtained an LP extract with 6.07 mg/g of RA. However, this extract does not report the presence of salvianolic acid B.

On the other hand, luteolin (4.98 mg/g DE) and apigenin (2.74 mg/g DE) were also reported in this LP extract. However, the concentration is lower than obtained in our study with CE and green techniques. In agreement with our results, Costa et al. [30] reported CGAs (0.47 mg/g DE) however, the LP extract obtained with MAE presents a total of CGAs of 5.1 mg/g DE. While Mansinhos et al. [4] reported a few traces of CGA in the UAE extract, in our study, about 2.74 mg/g DE of CGAs are reported. In contrast, the ferulic acid content in this study is six times higher than that obtained in the UAE extract (0.50 ± 0.07 mg/g DE). The achieved variability in phenolic quantification could have been attributed to several factors. The absence of optimization parameters for each extraction method might be the most relevant. Additional factors might include different parameters in the techniques such as power, temperature, or solvent composition, and in addition, the use of other regional LP varieties and gathering conditions.

According to the HPLC-DAD analysis, CET showed the highest content of phenolic compounds (173.05 mg/g DE). Moreover, for the UAE method, the total phenolic compounds by HPLC are lower (157.31 mg/g DE) than MAE (163.53 mg/g DE). The same tendency was observed in the TPC assay, although those differences were not statistically significant *(p* < 0.05) in this assay. In agreement with this, a recent study aiming to improve the extraction of RA from Lamiaceae herbs showed that MAE and CE methods result in similar yields [38]. Based on this, it can be concluded that MAE is only superior to CE in relation to extraction time. In addition, with HPLC analysis, it was possible to identify that although MAE presents similar levels of TPC, its flavonoid content is much higher than CE. Overall, the differences observed between studies could be explained by the geographical area influencing the chemical composition, namely the edaphoclimatic factors. Soil microbiota, climacteric environment, air humidity, and daily sun exposure are established by the scientific community as primarily responsible for the changes in phenolic compounds observed in plants of the same species.

### 3.4. Antioxidant Activity

**Figure 2:**
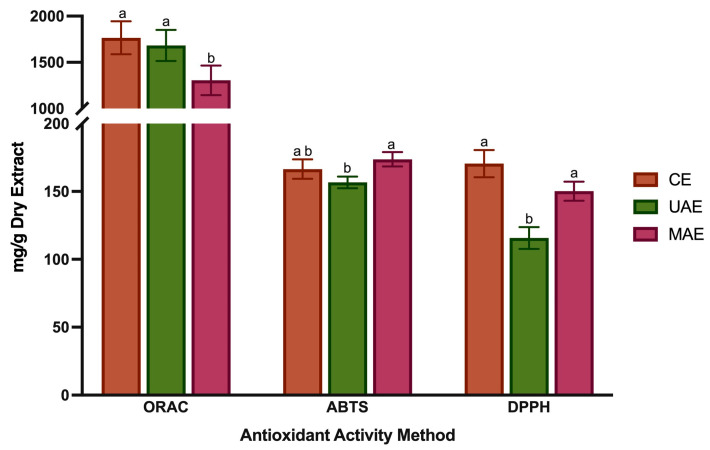
Antioxidant activity of LP extracts by ORAC method (expressed as mg TE/g DE), ABTS method (expressed as mg AAE/g DE) and DPPH method (expressed as mg TE/g DE). Values are represented by the average ± standard deviation. Different letters (a,b) mean significant differences between extraction methods (*p* < 0.05).

The use of plant-based ingredients and extracts in the food industry has grown due to concerns about the potential adverse effects of synthetic antioxidants. Studies have indicated that natural antioxidants are associated with a lower risk of non-communicable diseases, making them a viable replacement for synthetic antioxidants in food products. *Lavandula* species are recognized for their natural antioxidant properties, which have biological significance and offer economic benefits, mainly when extracted from under-utilized plants like LP. The antioxidant activity was evaluated using three different in vitro assays—ABTS, DPPH and ORAC—and the results are shown in Figure 2. The antioxidant activity measured by ABTS ranged from 156.7 ± 4.3 to 166.5 ± 7.2 mg AAE/g DE, and no statistically significant differences were found between the CE and MAE extraction methods (*p* > 0.05). The DPPH values ranged from 115.7 ± 12.0 to 170.5 ± 12.1 mg TE/g DE, with the lowest value belonging to UAE and the highest to CET and MAE (*p* < 0.05). The radical scavenging effects measured by the ORAC assay ranged from 1306.0 ± 159.8 to 1765.5 ± 214.3, mg TE/g DE, with the lowest value belonging to MAE and the highest to CET and UAE (*p* < 0.05). As shown, the values obtained by the ORAC method were 10-fold higher than those obtained by ABTS and DPPH methods. These differences probably result from the different mechanisms, single electron transfer and hydrogen atom transfer, involved during DPPH/ABTS and ORAC assays. Additionally, DPPH was dissolved in methanol, while ABTS was performed under an aqueous solution; therefore, the DPPH method results in the aprotic reaction being better adapted for determining the effects of less-polar phenolic compounds such as flavonoids. This explains why the extract obtained with MAE presents higher values than the ones obtained with CE and UAE in the DPPH method. This extract has more flavonoids (Section 3.2). In addition, the values of DPPH revealed an excellent correlation (r^2^ = 0.898) with the flavonoid content.

These discrepancies between the obtained values show why combining more than one method is important to determine the in vitro antioxidant capacity. LP polyphenol-rich extracts obtained by CET showed the highest antioxidant capacity on ORAC assay. These values are consistent with the total phenolic content from HPLC-DAD analysis, which indicates that phenolic compounds are important contributors to the antioxidant properties in LP. Compared to MAE, the high antiradical activity of this extract could be partially attributed to the presence, in the highest amounts, of RA, a compound known for its high redox potential. Among all bioactivities, the antioxidant activity is the most studied. Studies from Baptista et al. [33] have shown that when tested at 50 μg/mL, the methanolic extract from LP showed DPPH radical scavenging inhibition value of about 60%., while the extract obtained with water at the same concentration showed an inhibition percentage of approximately 15%. Curiously, the aqueous extracts only exhibited significant DPPH radical scavenging activity values when tested at 400 μg/mL (68.9 ± 2.9%). Ferreira et al. [42] tested the ethanolic extracts and decoctions of LP using the DPPH method and the samples revealed antioxidant activity. Baptista et al. [33] also studied the antioxidant potential of different extracts from L. pedunculata, and all samples displayed a high DPPH scavenging activity. Moreover, Costa et al. [30] assessed the radical scavenging effects of the essential oil and polar extracts from *L. pedunculata* subsp. *lusitanica* by ABTS and ORAC assays. After the infusion method, the CE with ethanol 50% (*v*/*v*) without temperature was the most efficient at neutralizing ABTS and peroxyl radicals, while the essential oil was the weakest. Finally, a more recent study with LP extracts from UAE [4], also reported good results in the ABTS, DPPH and ORAC assays. Nevertheless, the results obtained in our study were more promising in terms of antioxidant potential since in ABTS and DPPH, the values range from 20 to 70 mgTE/g DE and in ORAC from 50 to 275 mgTE/g DE.

### 3.5. Antimicrobial Activity

**Figure 3:**
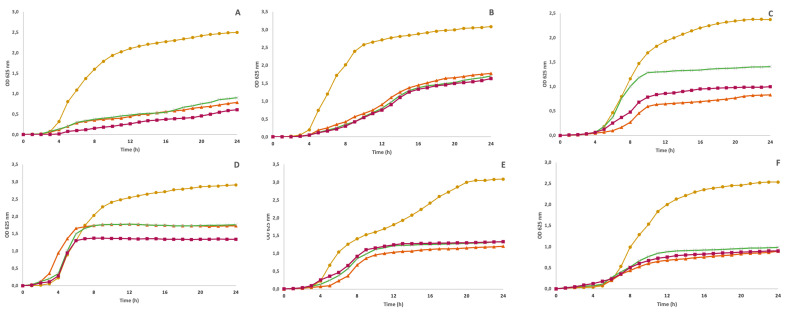
Growth inhibition curves by optical densities (at 625 nm) of *Bacillus cereus* (**A**), *Listeria monocytogens* (**B**), *Staphylococcus aureus* (**C**), *Escherichia coli* (**D**), *Pseudomonas aeruginosa* (**E**) and *Salmonella enterica* (**F**) when incubated with different LP polyphenol-rich extracts extracted based on CE (▲, in orange), UAE (✖, in green) and MAE (◼, in pink) at 10 mg/mL and positive control (●, in yellow).

The antibacterial activity of LP polyphenol-rich extracts was tested against a panel of six bacteria, including three Gram-positive bacteria (*S. aureus*, *B. cereus*, and *L. monocytogenes*) and three Gram-negative bacteria (*E. coli*, *S. enterica*, and *P. aeruginosa*), specifically selected basis on their importance to public health. Figure 3 presents the growth inhibition curves obtained for each LP extract at 10 mg/mL (highest concentration tested) and Table 5 shows the growth inhibition (%) after 24 h exposure to LP polyphenol-rich extracts. Overall, the LP extract presents good bacterial growth inhibition at 10 mg/mL regardless of the extraction method. However, no bactericide concentration was detected in our study. Conversely, the work of Lopes et al. [3] demonstrated that the MIC/MBC with LP hydroethanolic extracts for *B. cereus*, *S. aureus*, *L. monocytogenes*, *E. coli* and *P. aeruginosa* was 0.075/015, 0.45/0.6, 0.45/0.6, 0.2/0.3 and 0.3/0.45 mg/mL for LP growth in Portugal, respectively. While the study indicates significantly greater antimicrobial activity than our LP extracts, the discrepancy in phenolic compound content suggests that the variance in antimicrobial results could be attributed to the analytical method.

As shown, the least resistant microorganisms were *B. cereus*, *P aeruginosa* and *L. monocytogenes* since they were inhibited by all extracts regardless of the concentration used. However, the greatest inhibition occurs at the highest concentration (10 mg/mL) with MAE for *B. cereus* (*p* < 0.05), while for *L. monocytogenes*, no statistical differences were found between the CE and MAE extract. In addition, regardless of the extraction method, at 10 mg/mL, all extracts demonstrated the same inhibition capacity for *P. aeruginosa* (*p* > 0.05). The extract obtained with MAE showed higher inhibition for both Gram-positive bacteria (*B. cereus* and *L. monocytogenes*) and all Gram-negative bacteria. Meanwhile, the LP extract obtained with UAE showed a lower inhibition, which agrees with the lower TPC present in this extract and antioxidant activity. Phenolic compounds and other natural agents such as EOs showed different antimicrobial mechanisms of action; however, the mechanism that is mainly reported is interference on the cell membrane including changes in their structure and function, which may affect electron chain transport, enzyme activity, nutrient uptake and synthesis of nucleic acids and proteins [43].

On the other hand, the most resistant bacteria was *E. coli* since only 10 mg/mL could inhibit 40.5, 39.4 and 59.0% of the growth with CE, UAE and MAE extracts, respectively. Gram-positive bacteria are generally more susceptible because the cell wall has a thick peptidoglycan layer. In contrast, Gram-negative bacteria have a thin peptidoglycan layer and an extra layer, an outer membrane, that consists of phospholipids and lipopolysaccharides [44]. Despite *P. aeruginosa* showing outstanding inhibition with LP extracts, the results obtained in this study agree with those findings in the literature. However, experiments with pure phenolic compounds showed that phenolic acids, such as RA, chlorogenic acid, caffeic acid and some flavonoids (quercetin, luteolin-7-*O*-glucoside and apigenin-7-*O*-glucoside), may be effective against both Gram-negative and Gram-positive bacteria [45]. The presence of caffeic acids in our extracts may explain why *P. aeruginosa* was also inhibited with lower extracts concentration. It is known that a high content of hydroxycinnamic acid and flavonoids is correlated with antioxidant and antimicrobial activities [45,46]. According to our findings from the HPLC-DAD analysis, the substantial presence of identified phenolic compounds, such as RA, chlorogenic acid and caffeic acid in *Lavandula* extracts obtained with the MAE and CE methods, could be the main reason for the antibacterial capacity of these extracts. RA is a caffeic acid ester found in different plants of the Lamiaceae family with antioxidant, anti-inflammatory, and antibacterial effects. Chlorogenic and caffeic acids have also been reported as compounds with significant antioxidant and antimicrobial activity [3,45,47]. However, both CE and MAE extracts presented similar antibacterial activity despite the differences in the average content of each phenolic identified in both extracts. This may happen because of the additive and synergistic effects of phytochemicals in the extracts [45]. Overall, the production of LP extracts with MAE has several utilities and applications in different fields, such as food preservation to replace synthetic additives and to extend the shelf-life of perishable foodstuffs.

### 3.6. Tyrosinase Inhibition

**Figure 4:**
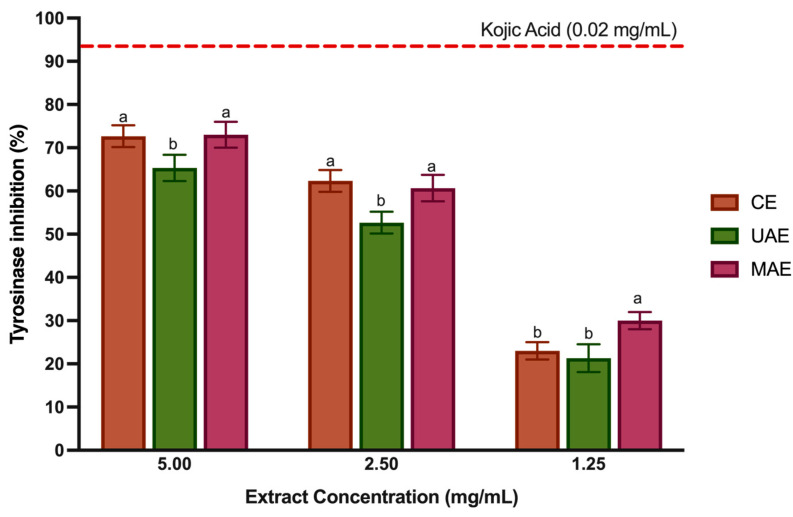
Tyrosinase inhibition (%) for the LP polyphenol-rich extracts at different concentrations (5, 2.5 and 1.25 mg/mL). Values are represented by the average ± standard deviation. Different letters (a,b) mean significant differences between extraction techniques for the same tested concentration (*p* < 0.05).

Tyrosinase is copper-containing enzyme, also known as PPO, found in mammals, bacteria, fungi and plants, which controls the production of melanin and catalyzes the hydroxylation of monophenols to o-diphenols and their subsequent oxidation to *o*-quinones [48]. Tyrosinase has been implicated in skin diseases and esthetic characteristics such as freckles, melasma, age spots, and in Parkinson’s and Huntington’s diseases [4]. In addition, this enzyme is responsible for enzymatic browning reactions in fruits and vegetables [37]. Therefore, in the food industry, tyrosinase inhibition is key, as the enzymatic browning contributes to undesirable oxidation in fruit and vegetables, which causes unwelcome effects on their safety, organoleptic properties, and nutrition [49]. Phenolic compounds can interfere with the production of browning catalyzed by tyrosine via (i) reducing the products of the reaction (quinones) and (ii) inhibiting the tyrosinase activity. Therefore, many tyrosinase inhibitors based on polyphenol-rich extracts have been discussed, but only a few have possessed enough potency and safety for food industry application [48]. Hence, pursuing novel, safe, and efficient tyrosinase inhibitors has emerged as an appealing objective. To the best of our knowledge, only two research studies were found concerning the capability of LP polar extracts (obtained with UAE and with CE [4,35]) to inhibit tyrosinase activity. Therefore, this study reports for the first time tyrosinase inhibition with LP extracts obtained with MAE. Figure 4 displays the results for the tyrosinase inhibitory potential of LP extracts. Kojic acid and arbutin are a natural tyrosinase inhibitor clinically used to cure human hyperpigmentation and prevent browning in food products [50]. Thus, this study used kojic acid as a positive control, at 0.02 mg/mL showed a capacity to inhibit tyrosinase of 93.4%. LP polyphenol-rich extracts (5–1.25 mg/mL) could inhibit tyrosinase between 73.01% and 21.33%. However, the inhibition is not directly dependent on extract concentration. The CE and MAE showed better enzyme inhibition at 2.5 mg/mL (*p* < 0.05). However, despite lower inhibition (30.13%) at 1.25 mg/mL, the MAE extract demonstrated greater tyrosinase inhibition capacity than the other extracts (*p* < 0.05).

The big difference between the extracts obtained with MAE and CE is in the number of flavonoids. The structure of flavonoids is compatible with the competitive inhibition of tyrosinase, and detailed studies have shown that some flavonoids such as luteolin, apigenin and quercetin are quite potent inhibitors, some of them with a lower IC_50_ than kojic acid [51]. Otherwise, regardless of tested concentration, the UAE extract showed the lowest % inhibition (*p* < 0.05). This is explained by the fact that this extract generally has fewer phenolic compounds. In agreement, Mansinhos et al. [4] also detected less enzyme inhibition with the LP extract obtained by UAE with ethanol 80% (*v*/*v*) than in the extracts obtained with DES. In addition, Zengin et al. investigated the capacity of water extracts from LP as tyrosinase inhibitors and showed a good inhibitory capacity, which agrees with our results.

Isolated compounds in high amounts in LP extracts showed a good ability to inhibit tyrosinase. In a prior study, Kang et al. [52] demonstrated the inhibitory potential of RA against mushroom tyrosinase, achieving an IC_50_ value of 16.8 µM. Notably, they observed that the bioactivity of RA was comparable to that of kojic acid, with IC_50_ values of 22.4 µM. Regarding hydroxycinnamic acids, they were shown to have a dual role, functioning either as tyrosinase inhibitors or as enzyme substrates. Furthermore, studies showed the activity of diCGAs were twice as effective as those of CQAs [53]. Likewise, some natural extracts rich in ferulic acid also have strong anti-tyrosinase activity [54]. Furthermore, combining various phenolic compounds effectively inhibits tyrosinase activity rather than employing individual compounds.

As previously mentioned, enzymatic browning represents a significant challenge in the food industry, especially for IV gamma products. To prevent this, natural food additives based on LP polyphenol-rich extract could replace synthetic compounds such as ascorbic acid and sulfite-containing compounds.

### 3.7. α-Glucosidase and ACE Inhibition

As per the World Health Organization (WHO), metabolic syndrome, characterized by elements like obesity, insulin resistance, and hypertension, stands as a significant health threat in the modern era. Among these, hypertension and type 2 diabetes are considered leading risk factors. Newly released data indicate that approximately 425 million individuals globally are currently living with diabetes, with a potential increase of 48% projected by 2045 [55]. The population affected by hypertension is estimated to be 1 billion, contributing to an annual mortality of around 9.4 million people worldwide [56]. Numerous studies have supported the pharmacological properties of plants in managing diabetes and hypertension, presenting them as a viable alternative in healthcare [57]. The antihypertensive potential of LP polyphenol-rich extracts remains unexplored, regardless of the extraction method used. Previous research has extensively examined ACE inhibitory properties in *Lavandula* genus compounds like EOs [58]. ACE is a metalloproteinase that regulates blood pressure by converting angiotensin I into angiotensin II, a potent vasoconstrictor. In addition, ACE can degrade bradykinin (BK), a potent endogenous peptide vasodilator. ACE inhibition enhances the effects of vasodilator bradykinin (BK) and reduces angiotensin II formation, explaining the benefits of ACE inhibition [59]. The traditional antihypertensive drugs such as captopril and lisinopril showed several side effects [60]. Hence, there is growing interest in discovering safe and efficient ACE inhibitory compounds from plants. The IC_50_ results of CE, MAE and UAE extract were 1.06, 0.98 and 1.17 mg extract/mL, respectively, as illustrated in Table 6. The results can also be expressed based on the TPC, thus demonstrating that extracts with the highest content of phenolic compounds have the lowest IC_50_ (MAE and CE). The specific mechanisms responsible for this enzyme inhibition remain unknown. Still, some of the cardiovascular effects produced by RA, the main phenolic acid present in the LP extracts, include ACE inhibition and/or modulation. For this reason, some reports associated this compound with significant reductions in arterial pressure [59].

In addition, the bradykinin dose–response curves showed that RA promoted a systolic blood pressure reduction similar to captopril. Salvianolic acid B also presents a huge quantity in the extracts, impacting ACE inhibition. Ye et al. [61], using molecular docking methods, found that salvianolic acid B affected ACE and renin to relax blood vessels and regulate hypertension. Other phenolic compounds can inhibit ACE activity and reduce blood pressure. Indeed, using in vivo assays with spontaneous hypertensive rats, blood pressure lowering effects have been confirmed for quercetin and ferulic acid [62]. The phenolic acids and flavonoids are reported to inhibit ACE activity via interaction with the zinc ion on the active site. In addition, the significance of hydroxyl and methoxy groups for zinc metalloproteinase inhibition is also reported [15]. The presence of specific functional groups, such as hydroxyl seems to increase the potency to inhibit ACE as these can act as hydrogen bond acceptor or donor. Conversely, the presence of methoxy groups negatively influences ACE inhibitory activity. Regarding the flavonoids, some studies reported the importance of the hydroxy group on position 7 in the structure of flavonoids for inhibiting ACE enzyme activity [15]. Therefore, the presence of caffeic acid, luteolin-7-*O*-glucoside and apigenin-7-*O*-glucoside could be important for the significant antihypertensive activity observed in the extracts.

However, ACE activity inhibition is not solely attributed to phenolic compounds. This study did not assess triterpenoids and small peptides, recognized for their ACE inhibitor capabilities [63]. To our knowledge, this is the first time that iACE activity in different LP polyphenol-rich extracts was investigated. Therefore, the extracts developed from this medicinal plant serve as an natural alternative as a nutraceutical promoting the cardiovascular health of the population.

**Figure 5:**
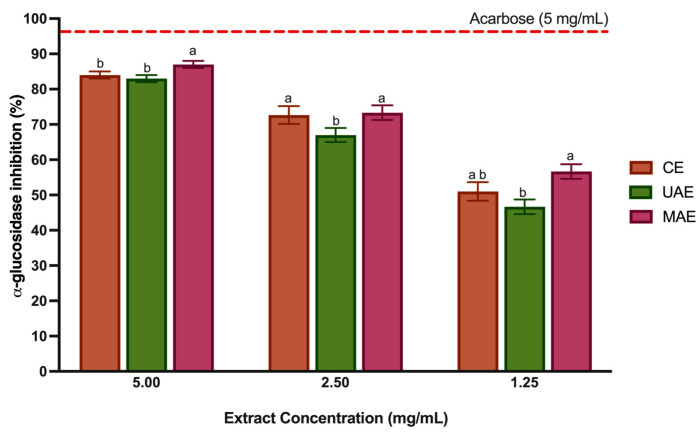
Antidiabetic activity (α-glucosidase inhibition assay) of LP polyphenol-rich extracts. Values are represented by the average ± standard deviation. Different letters (a,b mean significant differences between extraction techniques for the same tested concentration (*p* < 0.05).

Over the past decade, there has been an increase in the number of publications studying the effect of polyphenols on diabetes. All classes of phenolic compounds have shown antidiabetic potential [64]. The research suggests that the primary mechanism of action is associated with inhibiting glucose absorption by inhibiting digestive enzymes such as α-glucosidase and α-amylase [7,65,66]. In the small intestinal epithelial cell, α-glucosidase is an oligosaccharide hydrolase responsible for breaking down oligosaccharides and disaccharides into monosaccharides. Inhibiting the activity of α-glucosidase can slow down carbohydrate absorption, effectively regulating postprandial hyperglycemia and aiding in managing type 2 diabetes [37]. Some in vitro and in vivo studies have reported antidiabetic activity through enzyme inhibition in LP extracts and other *Lavandula* species, obtained through CE with water and methanol [7,35,67]. However, the bioactivity of LP extracts related to α-glucosidase inhibition or other mechanisms has never been studied with LP extracts obtained through green techniques such as UAE and MAE. In addition, to the best of our knowledge, it is the first time that LP of Portuguese geographical origin has been tested for α-glucosidase inhibition. Figure 5 displays the results of the α-glucosidase inhibition by the LP extracts at three different concentrations (5, 2.5 and 1.25 mg/mL) and acarbose, a positive control. Acarbose is the most frequently used drug for diabetes and, in most studies, it has been shown to increase the life expectancy in type 2 diabetes patients. In some cases, a variety of severe side effects such as abdominal pain, diarrhea, flatulence, and skin problems can also occur [68]. The three polyphenol-rich extracts, independent of concentration, showed higher α-glucosidase inhibition. However, as the extract concentration decreases, the % inhibition decreases, even if not directly proportional. At 5 mg/mL, the LP polyphenol-rich extracts and the commercial drug (acarbose) showed a similar activity; however, the MAE extracts presented a higher activity than the remaining extracts (*p* < 0.05). Furthermore, as the tested concentration decreased, this extract and the CE extract remained the most significantly active (*p* < 0.05) in inhibiting the enzyme. These results agree that these two extracts display a higher content of phenolic compounds than the extract obtained with UAE. In particular, the MAE extract showed a higher amount of flavonoids (Section 3.3), which have been reported by Ali et al. [69] using molecular docking techniques to have a significant role in the α-glucosidase inhibition. Despite differences in extraction techniques and solvents, our results agree with studies reported with extracts with LP from Morocco and Turkey. The LP extracts obtained in this studies (CE with methanol) showed considerable α-glucosidase inhibition in vitro [35] and acute and chronic oral administration of LP aqueous extract reduced the peak of the glucose concentration [7]. Several important hydroxycinnamic acids present in high concentration in LP extracts, such as ferulic acid [70], chlorogenic acid [71,72], caffeic acid [73] and RA [74,75] have shown considerable hypoglycemic activity via in vitro and in vivo experiments. The potential interaction mechanism between these BCs and α-glucosidase reported in the literature describes the formation of hydrogen bonds or hydrophobic forces in the enzyme’s active site [70]. In particular, the in vitro studies of Kubínova et al. [74] showed that IC_50_ of RA was four-times lower than acarbose. In addition, this phenolic acid has been demonstrated to regulate glucose homeostasis, restoring the blood glucose level and regulating adiponectin and leptin levels in diabetic rats [76,77]. Also, the salvianolic acid had a potent α-glucosidase inhibitory activity, whilst the interaction mechanisms remain unclear [68]. Other important flavonoids such as apigenin and luteolin present in LP extracts are reported for their capacity to inhibit α-glucosidase competitively, forming complexes where the main forces driving the interaction were hydrophobic and hydrogen bonding [78].In recent studies, gentisic acid showed a moderate antidiabetic activity by inhibiting α-glucosidase [79].

Although the values of α-glucosidase are inferior to those obtained for acarbose, the results show high potential. In addition, the LP polyphenol-rich extracts were obtained from a natural, unexplored source with green techniques and solvents, allowing the safe incorporation into food products. These extracts may be a sustainable alternative to regulate type 2 diabetes.

### 3.8. Cytotoxicity and Mutagenicity

**Figure 6:**
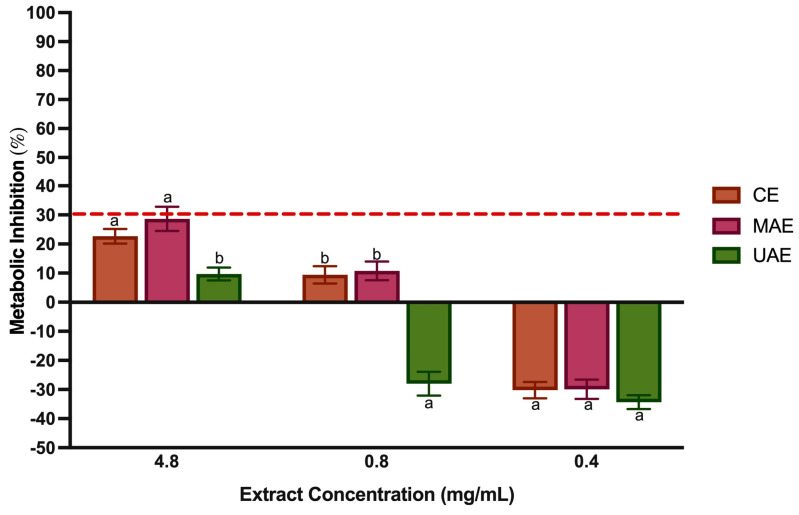
Cytotoxicity of LP polyphenol-rich extract at different concentrations. The dotted line represents the 30% cytotoxicity limit defined by the ISO 10993-5 [25]. Values are the mean ± standard deviation. Different letters mean significant differences (*p* < 0.05).

The human epithelial cell line caco-2 monolayers have been widely used for mimicking intestinal conditions, providing a reliable model for investigating the absorption of BCs after oral administration in humans [80]. They are routinely used to determine the mechanism of action of BCs or extracts to move towards preclinical animal studies and human clinical trials for ensuring the safety of the final food product [81]. As shown in Figure 6, LP extracts did not exert any inhibitory activity upon caco-2 cellular metabolism at all tested concentrations. This is in line with the standard ISO 10993-5:2009 [25], which considers as cytotoxic when the cell viability is lower than 70%. However, the MAE extract at 4.8 mg/mL had a value of 28.67 ± 4.16%, which is very close to the limit established as toxic.

In contrast, the UAE extract at the same concentration showed about 3-fold less metabolic inhibition (*p* < 0.05). Interestingly, when considering the impact of the lowest LP concentration upon caco-2 metabolism, an apparent metabolic stimulation (metabolism inhibition negative values) represents a metabolic activity higher than the growth control. Considering this result, it is recommended to quantify the DNA using Pico Green Assay, as performed by Rodrigues et al. [82], to assess the actual effect of LP extracts on metabolic activity.

As far as we know, no cytotoxicity studies on caco-2 cells exist in the literature with LP extracts. However, Costa et al. [83] studied the cytotoxicity of *Lavandula viridis* L’Hér (*Lavandula* genus) extract at 0.5 mg/mL obtained under CE with water:ethanol 50% (*v*/*v*) and showed that the viability of the caco-2 cell line was not affected by the extracts. The same study also proved no metabolic inhibition for RA (at concentrations of 0.125 and 0.250 mg/mL), the main phenolic compound in *Lavandula viridis* L’Hér. [75].

Evaluating genotoxic properties is an essential step of the safety assessment of substances intended to be used as pharmaceuticals, nutraceuticals, or food additives. The Ames test was standardized in the 1975s by Ames et al. [84] to evaluate the mutagenic capabilities of chemicals. Recently, the Ames test has become a commonly employed method for identifying both the mutagenic and antimutagenic properties of medicinal plants and extracts derived from plants. The Ames test uses *Salmonella typhimurium* strains that are auxotrophic for histidine to detect gene mutations, specifically as base pair substitutions and frameshift mutations. Moreover, it is emphasized how crucial it is to conduct the Ames test both in the absence and presence of the exogenous metabolic system (S9). The addition of the S9 fraction is essential to replicate the metabolism of the test substance as it would occur in mammals. Consequently, tests lacking these two conditions jeopardize the accuracy of findings and compromise the safety assessment of the extracts under evaluation.

In the genotoxic profile of the polyphenol-rich LP extracts (Table 7), no harmful effects were found, as none of the tested concentrations exerted any genotoxic effects, either with and or without metabolic activation, against the tested strain, according to parameters defined by the manufacturer of the Ames test kit and the OECD guidelines. Briefly, to conclude the genotoxicity, the values should be at least 2.5 times higher than those obtained with the solvent control (water in this study). So, with S9, the values should be lower than 28.75; without S9 activation, the values should be lower than 41.25. To our knowledge, this paper reported for the first time the genotoxicity of LP extracts whether obtained using the CE methodology or via green technologies such as MAE and UAE.

The absence of cytotoxicity and mutagenicity in the extracts suggests that the present components did not induce cellular or DNA damage. Therefore, they can be considered safe at the concentrations tested. Among the extraction methods employed, UAE emerged as the safest choice due to its slightly lower phenolic compound content.

## 4. Conclusions

To achieve a change encouraging the conservation and valorization of Portuguese flora, this research article highlights important insights concerning evidence of the bioactivity of LP polyphenol-rich extracts obtained with different extraction methodologies (CE, MAE, and UAE) such as antioxidant, antimicrobial, anti-hypertensive, antidiabetic and anti-browning effects, as well as proving the safe use for human consumption through cytotoxicity and mutagenicity assays.

The findings demonstrate that MAE is a superior method in terms of the time and energy saved compared to UAE; however, the extraction yields and phenolic compounds obtained in the extracts from *LP* are very similar to those obtained through CE. MAE enabled the extraction of a comparable amount of TPC to CE but in a shorter extraction time, reducing energy consumption. Both polyphenol-rich extracts exhibited higher quantities of RA, salvianolic acid, 4,5-Di-*O*-caffeoylquinic acid and luteolin-7-*O*-glucoside; therefore, demonstrated increased antioxidant capacity evaluated by in vitro methods. In addition, the extracts showed great growth inhibition for *B. cereus*, *S. aureus*, *S. enterica* and *P. aeruginosa* (>50%) at very low concentrations (10 mg/mL). As we understand it, the significant positive antihypertensive and antidiabetic activity in LP extracts are mainly obtained with MAE; this extract showed potent antihypertensive activity (IC_50_ = 0.98 mg DE/mL), and antidiabetic activity (87% at 5 mg/mL) was reported for the first time. Moreover, the same extract also showed significant tyrosinase inhibition (73% at 5 mg/mL). Ultimately, the extracts demonstrated no cytotoxic or genotoxic effects, irrespective of the extraction method, affirming their safe application within the food industry, particularly as antioxidant and antimicrobial food additives. Additionally, this underscores the substantial potential to utilize LP extracts in the production of nutraceutical products, offering antihypertensive and antidiabetic effects.

Overall, the results and evidence will be the beginning of a possible increase in the exploitation and valorization of LP, which may trigger its major production and industrial processing to develop and optimize sustainable food-based products. Explored Portuguese plants bring value to the country while minimizing environmental impact by using other important raw materials to produce natural functional ingredients, promoting the bioeconomy.

## Figures and Tables

**Table 1:** Extractive yields (*w*/*w*, %) obtained by the different extraction methods (CE, UAE and MAE) from LP. Values are represented by the average ± standard deviation. Different letters mean significant differences between extractions (*p* < 0.05).

Matrix	Extraction Method	Extractive Yield (%)
*Lavandula pedunculata*	CE	23.91 ± 2.00 ^a^
	UAE	16.17 ± 2.81 ^b^
	MAE	17.64 ± 1.63 ^b^

**Table 2:** The total phenolic (TPC) and total flavonoid content in LP polyphenol-rich extracts. Values are represented by the average ± standard deviation. Different letters mean significant differences between extraction methods (*p* < 0.05).

**Matrix**	**Extraction Method**	**TPC** **(mg GAE/g DE)**
*Lavandula pedunculata*	CE	183.7 ± 17.8 ^a^
	UAE	181.4 ± 6.5 ^a^
	MAE	183.1 ± 4.9 ^a^

**Table 3:** LC-ESI-UHR-QqTOF-MS data of organic acids and phenolic compounds identified in CE, MAE and UAE in LP polyphenol-rich extracts.

Proposed Name	Molecular Formula	Rt	m/z Measured Mass [M-H]^−^	MS^2^ Fragments (m/z, % Base Peak Intensity)	Error (mDa)
Gluconic Acid	C_6_H_12_O_7_	1.36	195.0510	75 (100)	1.0
Tartaric acid	C_4_H_6_O_6_	1.5	149.0092	72 (100)	0.1
2-Furoic acid	C_5_H_4_O_3_	2.2	111.0088	69 (100)	0.5
Succinic acid	C_4_H_6_O_4_	2.8	117.0193	73 (100)	−0.3
Malic acid	C_4_H_6_O_5_	1.7	133.0143	71 (100)	0.3
Citric acid	C_6_H_8_O_7_	2.1	191.0197	87 (100), 111 (39)	0.8
Azelaic acid	C_9_H_16_O_4_	13.2	187.0975	97 (100), 125 (66)	0.9
3-*O*-caffeoylquinic acid	C_16_H18O_9_	6.4	353.0878	191 (100), 179 (63), 135 (25),	0.7
5-*O*-caffeoylquinic acid	C_16_H_18_O_9_	8.2	353.0878	191 (100), 173 (97), 179 (80)	1.1
4-*O*-Caffeoylquinic acid	C_16_H_18_O_9_	8.9	353.0887	173 (100), 179 (80), 191 (62), 135 (21)	1.0
1,5-Di-*O*-caffeoylquinic acid	C_25_H_24_O_12_	10.1	515.3949	163 (98), 353 (20)	0.8
4,5-Di-*O*-caffeoylquinic acid	C_25_H_24_O_12_	12.9	515.1008	353 (100), 173 (80), 179 (40)	0.9
Caffeic acid	C_9_H_8_O_4_	8.5	179.0351	135 (100), 179 (40)	1.4
Isoferulic acid	C_10_H_10_O_4_	8.6	193.0506	134 (100)	1.0
Ferulic acid	C_10_H_10_O_4_	11.8	193.0506	134 (100), 178 (74), 193 (34)	0.7
*p*-coumaric acid	C_9_H_8_O_3_	10.4	163.0401	119 (100), 163 (20)	−0.6
Lithospermic acid A	C_27_H_21_O_12_	13.9	537.1038	359 (100), 295 (80), 197 (42), 179 (50), 493 (18), 313 (10)	1.8
Rosmarinic acid	C_18_H_15_O_9_	13.6	359.0772	161 (100), 197 (60), 179 (54)	1.5
Salvianolic acid A	C_26_H_22_O_10_	13.9	493.1141	185 (68), 295 (100)	−2
Sagerinic acid	C_36_H_32_0_16_	13.6	719.1684	161 (100), 359 (80), 197 (20), 179 (11)	1.9
Salvianolic acid B	C_36_H_30_O_16_	14.8	717.1520	537 (50), 519 (40), 339 (8), 321 (100), 197 (6), 179 (27)	1.9
trans-4-Hydroxycinnamate	C_9_H_8_O_3_	9.1	163.0401	119 (100)	0.6
1-*O*-Vanilloyl-beta-D-glucose	C_14_H_17_O_9_	5.7	329.0878	167 (100)	0.1
Protocatechuic acid	C_7_H_6_O_4_	5.7	153.0193	109 (100)	0.1
2,5-Dihydroxybenzoic acid	C_7_H_6_O_4_	7.3	153.0193	109 (100), 81 (35), 53 (32)	0.3
3,4-Dihydroxybenzaldehyde	C_7_H_5_O_3_	6.9	137.0244	108 (100)	1.0
4-Hydroxybenzoate-*O*-glucoside	C_13_H_15_O_8_	7.2	299.0772	137 (100)	0.7
Vanillylmandelic acid	C_9_H_10_O_5_	5.3	197.0455	72 (100), 123 (55), 135 (60)	0
Apigenin-8-*O*-glucoside	C_21_H_20_O_10_	13.3	431.0984	341 (100), 268 (87), 311 (75)	0.7
Luteolin-7-*O*-glucoside	C_21_H_20_O_11_	11.9	447.0933	447 (20), 285 (100)	1.4
Luteolin	C_15_H_9_O_6_	16.5	285.0131	285 (100), 133 (85)	1.1
Quercetin 3-*O*-glucoside	C_21_H_20_O_12_	12.2	463.0882	301 (100)	0.1
Apigenin-7-*O*-glucuronide	C_21_H_17_O_11_	13.4	445.0345	269 (100)	1.2
Luteolin-7-*O*-glucuronide	C_21_H_18_O_12_	12.1	461.9984	285 (100)	0.5
Apigenin-7-*O*-glucoside	C_21_H_20_O_10_	13.5	432.378	268 (100), 431 (20)	0.9
Apigenin	C_15_H_10_O_5_	17.6	269.0429	269 (100)	1.3
6-Hydroxyluteolin-7-glucoside	C_21_H_20_O_12_	11.7	463.0882	287 (100)	0.4

**Table 4:** Quantification (mg/g DW) via HPLC-DAD of phenolic compounds from LP polyphenol-rich extracts obtained by different extraction techniques. Values are represented by the average ± standard deviation. Different letters in the same row mean significant differences between extraction techniques (*p* < 0.05).

Phenolic Compound		CET	UAE	MAE
1	2,5-Dihydroxybenzoic acid	1.60 ± 0.06 ^a^	1.08 ± 0.09 ^b^	0.92 ± 0.03 ^c^
2	3-*O*-caffeoylquinic acid	0.48 ± 0.01 ^b^	0.43 ± 0.08 ^b^	1.46 ± 0.07 ^a^
3	5-*O*-caffeoylquinic acid	1.37 ± 0.17 ^b^	1.54 ± 0.11 ^b^	1.72 ± 0.05 ^a^
4	4-*O*-caffeoylquinic acid	1.56 ± 0.06 ^b^	0.77 ± 0.07 ^c^	1.92 ± 0.06 ^a^
5	Caffeic acid	0.31 ± 0.04 ^c^	0.92 ± 0.12 ^a^	0.66 ± 0.11 ^b^
6	1,5-Di-*O*-caffeoylquinic acid	7.13 ± 0.24 ^b^	7.86 ± 0.13 ^a^	7.83 ± 0.06 ^a^
7	Ferulic Acid	0.50 ± 0.09 ^b^	0.50 ± 0.07 ^b^	1.43 ± 0.06 ^a^
8	Luteolin-7-*O*-glucoside	12.79 ± 0.32 ^b^	10.82 ± 0.33 ^c^	17.56 ± 0.19 ^a^
9	Luteolin derivative *	6.04 ± 0.11 ^b^	5.12 ± 0.18 ^c^	6.53 ± 0.11 ^a^
10	4,5-Di-*O*-caffeoylquinic acid	28.52 ± 0.56 ^a^	25.25 ± 0.65 ^b^	19.28 ± 0.45 ^c^
11	Apigenin-7-*O*-glucoside	3.75± 0.19 ^b^	2.60 ± 0.15 ^c^	6.23 ± 0.12 ^a^
12	Apigenin derivative ^+^	0.87 ± 0.05 ^b^	0.84 ± 0.03 ^b^	1.40 ± 0.06 ^a^
13	Quercetin-3-*O*-glucoside	2.62 ± 0.18 ^c^	2.28 ± 0.07 ^b^	4.83 ± 0.10 ^a^
14	Rosmarinic acid	58.68 ± 1.42 ^a^	52.73 ± 1.86 ^b^	48.27 ± 1.92 ^c^
15	Salvianolic acid B	42.19 ± 0.71 ^ab^	40.09 ± 1.61 ^b^	43.19 ± 1.09 ^a^
16	Lithospermic acid ^#^	4.64 ± 0.24 ^b^	4.48 ± 0.34 ^b^	6.30 ± 0.16 ^a^
Total Phenolic Compounds		173.05	157.31	169.53
Total Phenolic Acids		146.98	135.65	132.98
Total Flavonoids		26.07	21.66	36.55

* quantified based on luteolin calibration curve; ^+^ quantified based on apigenin calibration curve; ^#^ quantified based on salvianolic acid B.

**Table 5:** Growth inhibition (%) for each pathogenic microorganism after 24 h exposure to LP polyphenol-rich extracts (CE, UAE, MAE) at different concentrations. Values are represented by the average ± standard deviation. Different letters (a–f) mean significant differences between the concentration tested (*p* < 0.05).

Microorganism		10 mg/mL			5 mg/mL			2.5 mg/mL		
		CE	UAE	MAE	CE	UAE	MAE	CE	UAE	MAE
Gram +	*Bacillus cereus*	68.6 ± 2.2 ^b^	64.0 ± 1.3 ^c^	75.8 ± 1.6 ^a^	63.6 ± 2.4 ^c^	48.8 ± 0.8 ^e^	69.0 ± 1.4 ^b^	56.5 ± 1.3 ^d^	20.5 ± 1.4 ^f^	50.2 ± 1.3 ^e^
	*Listeria monocytogenes*	47.1 ± 2.0 ^a^	43.7 ± 1.0 ^b^	47.1 ± 1.5 ^a^	42.5 ± 1.8 ^b^	38.1 ± 1.2 ^c^	41.0 ± 2.9 ^bc^	38.0 ± 2.0 ^c^	28.1 ± 1.3 ^d^	30.5 ± 0.9 ^d^
	*Staphylococcus aureus*	65.6 ± 2.4 ^a^	40.5 ± 2.1 ^d^	57.0 ± 2.3 ^b^	48.4 ± 1.1 ^c^	32.6 ± 1.0 ^d^	43.5 ± 2.1 ^e^	ni	ni	ni
Gram −	*Escherichia coli*	40.5 ± 1.3 ^b^	39.4 ± 2.2 ^b^	59.0 ± 1.6 ^a^	ni	ni	ni	ni	ni	ni
	*Salmonella enterica*	65.0 ± 1.5 ^a^	61.3 ± 3.0 ^ab^	64.6 ± 1.2 ^a^	57.5 ± 1.4 ^b^	50.3 ± 0.8 ^c^	48.2 ± 1.2 ^d^	ni	ni	ni
	*Pseudomonas aeruginosa*	60.7 ± 2.0 ^a^	57.1 ± 1.7 ^ab^	57.6 ± 1.8 ^a^	55.6 ± 1.7 ^bc^	44.0 ± 1.2 ^d^	52.5 ± 1.4 ^c^	45.9 ± 1.2 ^d^	28.3 ± 0.7 ^f^	40.6 ± 1.4 ^e^

ni, no inhibition growth.

**Table 6:** Antihypertensive activity based on iACE assay. Values are represented by the average ± standard deviation. Different letters (a,b) mean significant differences between extraction techniques for the same tested concentration (*p* < 0.05).

Matrix	Extraction Method	iACE	
		IC_50_ (mg Phenolic Compounds/mL)	IC_50_ (mg Extract/mL)
*Lavandula pedunculata*	CE	0.19 ± 0.01 ^ab^	1.06 ± 0.05 ^b^
	UAE	0.21 ± 0.01 ^a^	1.17± 0.04 ^a^
	MAE	0.18 ± 0.02 ^b^	0.98 ± 0.05 ^b^

**Table 7:** Results obtained for LP polyphenol-rich extracts AMES genotoxicity assay against *S. typhymurium* TA98 with and without the metabolic activation (S9). All values represent the average number of positive (revertant) wells ± standard deviation. Different letters (a,b) mean significant differences between only the LP polyphenol-rich extracts (*p* < 0.05).

Sample Test	TA 98	
	With S9	Without S9
Solvent Control (water)	11.51 ± 0.50	16.50 ± 1.50
Positive Control	303.00 ± 12.50	481.50 ± 3.50
CE	18.00 ± 2.00 ^a^	27.50 ± 0.50 ^a^
MAE	13.50 ± 3.00 ^ab^	20.00 ± 1.00 ^b^
UAE	10.00 ± 2.50 ^b^	18.50 ± 2.00 ^b^

## Data Availability

All data generated or analyzed during this study are included in this published article.
